# States of Uncertainty, Risk–Benefit Assessment and Early Clinical Research: A Conceptual Investigation

**DOI:** 10.1007/s11948-022-00418-w

**Published:** 2022-12-13

**Authors:** Antje Schnarr, Marcel Mertz

**Affiliations:** Institute of Ethics, History and Philosophy of Medicine, Hannover Medical School, Carl-Neuberg-Str. 1, 30625 Hannover, Germany

**Keywords:** Uncertainty, Ambiguity, Risk–benefit-assessment, Bioethics, Early clinical research

## Abstract

It can be argued that there is an ethical requirement to classify correctly what is known and what is unknown in decision situations, especially in the context of biomedicine when risks and benefits have to be assessed. This is because other methods for assessing potential harms and benefits, decision logics and/or ethical principles may apply depending on the kind or degree of uncertainty. However, it is necessary to identify and describe the various epistemic states of uncertainty relevant to such estimates in the first place. Therefore, this paper aims to develop a category system of different epistemic states of uncertainty which, although not exclusively, is primarily intended to be applied to early clinical trials. It is formed on the basis—and various combinations—of three dimensions of uncertainty that represent certain parts of incomplete knowledge: outcome (type of event), probability (of outcome) and evaluation (assessment of outcome). Furthermore, it is argued that uncertainty can arise from three different sources (the structure of the object of research, the state of the evidence, or individual handling of the research and already existing knowledge). The categories developed are applied to actual examples from gene therapy and genome editing to illustrate that they can be helpful for a more precise definition of the respective uncertainties, especially in the context of risk–benefit assessment. The categories allow a differentiated perspective of decision-making situations from the point of view of incomplete knowledge in general, but particularly, for example, in early clinical research, and may thereby support a more acceptable ethical assessment of potential harms and benefits.

## Introduction

### Ethical Importance of Acknowledging Uncertainty in Early Clinical Research (and Beyond)

Human actions and decisions, particularly in healthcare, can often mitigate suffering. However, our *ethical judgment* of harms and benefits in the biomedical context is dependent on our *knowledge* of these possible harms and benefits: “When investigators overestimate the probability of favorable outcomes, they potentially expose individuals to unjustified burdens, which may be considerable for phase 1 studies involving unproven drugs” (Kimmelman & London, 2011, p. 1). Whether an early clinical trial or a first-in-human trial should be conducted at all is based on existing preclinical evidence. This knowledge is often incomplete, which implies that decisions have to be made in states of *epistemic uncertainty*. In such a scenario, the concept of risk and how it is understood becomes important (Renn et al., 2011).

As Rudra and Lenk conclude based on systematic literature searches, in the context of clinical trials, an understanding of risk is usually shared that can more or less be summarized by the formula “risk = frequency × severity of damage” (Rudra & Lenk, 2021). There are also somewhat more complex suggestions for such formulas, e.g. “risk = frequency × [(kind of harm + magnitude (size + duration)]” (Rudra & Lenk, 2021, citing an example from King & Churchill, 2008). Still, two observations prevail: First, the usual risk–benefit assessment (RBA) in clinical trials is mostly oriented to rely on *known* probabilities or perceived likelihoods, which, however, becomes methodologically and ethically challenging in early phase trials (e.g. Genske & Engel-Glatter, 2016). This is because many early phase-trials are riddled with a *higher level* of uncertainty which goes beyond the uncertainty encountered in, for example, phase III efficacy trials (Kimmelman, 2012). Second, such an understanding of risk already presupposes (at least) *two* areas of knowledge where uncertainties can play a role, namely the aforementioned *probability* of an event occurring and the *evaluation* of this event. However, it is by no means inevitable that when an event occurs, it is always clear how it must be evaluated (and by whom), which is why it would be more appropriate to distinguish these two areas when it comes to the assessment of uncertainty. This all the more so if there is little or no experience with the type of event, as may well be the case with possible forms of harm (but also forms of benefit) in early clinical research—especially when new technologies are being used.

### The Example of Gene Therapy and Somatic Genome Editing

Gene therapy/somatic genome editing, including new methods such as CRISPR/Cas9 (Baumann, 2016), is a recent and striking example of epistemic uncertainty in early clinical research that utilizes new technologies. While ethical discussions about human germline editing are widespread (e.g. Halpern et al., 2019; Ishii, 2015; Ormond et al., 2017), ethical discussions on somatic genome editing are less so. This might be due to the fact that the main criteria in somatic genome editing are safety and efficacy issues (Hynes et al., 2017), which are probably considered *normal* issues for RBA, instead of the deeper social and ethical implications of editing germlines. Nonetheless, the example is well-suited to point out aspects of uncertainty that may have ethical implications precisely because such aspects are challenges for RBA.

In a recent publication, Bitterlinger et al. (2022) presented a ‘risk matrix’ intended for gene therapy and genome editing studies to various experts (clinical researchers, research ethics committee members, or members of national regulatory authorities), and interviewed them about the strengths and weaknesses of such a matrix as a tool for RBA especially in early human research. The ‘risk matrix’ was focused on so-called *mechanistic risk categories* of adverse reactions such as *insertional mutagenesis*, *carrier genotoxicity* or *(epi-)genetic instability* (see for details Bitterlinger et al., 2022); these are risks that, based on current evidence, could be expected when conducting gene therapy or genome editing studies. One of the results of this interview study was, however, that although some experts found such a “risk matrix” helpful in principle, they also made clear that it cannot say anything about those potential harms for which no or only scarce evidence is yet available—which cannot be ruled out in the case of such new methods as CRISPR/Cas9. In other words, such a ‘risk matrix’ is limited, as the name also suggests, to the assessment of possible risks (using a definition as outlined above), but leaves the field of uncertainty completely unaddressed. Decision-making under uncertainty is arguably increasingly moving away from the idea of a *calculable risk* that could be included in such a ‘risk matrix’. Uncertainty also complicates the drafting of informed consent forms and the subsequent provision of information to research participants (e.g. Kahrass et al., 2020), and is, generally spoken, also a major concern of research participants and potential patients themselves (e.g. Persaud et al., 2019; Wöhlke et al., 2019). However, there has been no attempt to date to classify uncertainty in such a way that allows creating a comparable “matrix” or “category system” for different types of uncertainty in early clinical studies.

Differentiating types of uncertainty—or *states of epistemic uncertainty*—is important as the respective uncertainty in a particular study may differ and, accordingly, the decisions and ethical judgments will (or even have to) differ as well. A category system of the epistemic states that are prevailing in such situations could, thus, support decision-making regarding whether it is acceptable to start first-in-human or early phase trials. In addition, when such trials are conducted, it could allow for a more accurate communication of the knowledge of potential harms and benefits, thus, also making informed consent processes more honest and transparent. Finally, it may help in the search for the causes of a particular state of uncertainty and the identification of adequate strategies to deal with given uncertainties (cf. Djulbegović, 2007).

### Developing a Category System of Different Epistemic States of Uncertainty

The need to distinguish different states of epistemic uncertainty is obviously not limited to early clinical trials, but is a rather general problem wherever (ethically sensitive) decisions have to be made in situations of uncertainty. Early clinical research is, thus, only one (albeit well-suited) instance of where this is especially relevant and, consequently, also a useful starting point to think about the more general epistemological topic of risk and uncertainty.

Understanding what is *meant* by ‘risk’, ‘uncertainty’ and other terms in the context of epistemic uncertainty is, however, not always easy. Different disciplines may use these terms in different ways (e.g. Althaus, 2005; Djulbegović, 2007; Ellsberg, 1961; Han et al., 2011; Möller et al., 2006; Stirling, 2010; Walker et al., 2013; Wynne, 1992). Therefore, the aim of this contribution is to clarify and consistently describe various epistemic states of uncertainty that can be especially relevant in clinical research contexts.

Although various (general) approaches to classify uncertainty exist (e.g. Bradley & Drechsler, 2014; Djulbegović, 2007; Han et al., 2011; Hansson, 1996; Walker et al., 2013), their classifications often have other goals. In particular, however, they have not been developed against the background of and with a view to concrete application in early clinical research. In classifications, the goals have clear implications on how a category system is constructed; so, epistemic states that are particularly important for clinical research contexts might differ from those relevant for other applications.^1^ Furthermore, theoretically, other classifications are not always systematically derived from, for example, basic knowledge states and their combinations, but are rather phenomenologically constructed (“as they face a decision maker”). Some approaches also characterize (some) uncertainties not via epistemic states, but rather via the *cause* for the particular uncertainty.

Accordingly, in the following, a *category system* for different states of uncertainty is developed. It is based on the assumption that specific states of uncertainty can be (logically) derived from the combinations of three so-called *dimensions of uncertainty* which represent what kind of knowledge (e.g. knowledge about the likelihood or about the outcome) is available or not (see below in detail). It is further based deliberately on the premise that uncertainties exist that are referred to as “great uncertainty” (Hansson, 1996) or “deep uncertainty”—uncertainties that can no longer be treated probabilistically (see e.g. Walker et al., 2013). There are problems (also in policy-setting) that “[…] cannot be dealt with through the use of probabilities and cannot be reduced by gathering more information, but are basically unknowable and unpredictable at the present time” (Walker et al., 2013, p. 399). There, it is especially debatable to what extent classical approaches of RBA are still applicable.^2^

Finally, the application of the category system, for example, to specific early clinical trials, may support the respective decision-making in that it allows a better identification of the specific RBA methods that are appropriate given a particular state of uncertainty. More importantly, it should also help to determine what type of decision logic and ethical principles/criteria are appropriate for ethical decision-making. The category system is, however, not limited to the application of early clinical trials, but is especially designed and, in some ways, also *tested* for use cases such as gene therapy/somatic genome editing (see “Approach”). This is because the category system can be understood as a basis for an extension of the ‘risk matrix’ already designed (see Bitterlinger et al., 2022), but directs the view to those epistemic states that were (and had to be) blanked out in this ‘risk matrix’.

## Approach

The term *uncertainty* was used as the basic term for describing states of insufficient or incomplete knowledge. It was relevant to separate such *epistemic states* of a person or collective of people clearly from the *causes* of these states. Subsequently, it was determined that a person or a collective of people could be in a state of uncertainty in four different ways. These were defined as the four possible *dimensions of states of uncertainty* (though one dimension was finally excluded for practical reasons, see footnote 8). A definition of *uncertainty* was provided on this basis. Specific states of uncertainty were understood as mere subtypes of the basic definition, and the similarities and differences between the specific epistemic states were determined by formalizing their conditions regarding the three dimensions via modal logic (see Table 1). Normal language descriptions and designations (*names*) of the specific epistemic states, such as ‘risk/chance’, ‘ambiguity’ and ‘indeterminacy’, were formulated or existing ones reworked.

**Table 1:** Specific states of uncertainty

State (common name in established literature)	Uncertainty dimensions	Formalization of uncertainty dimensions conditions (modal logic^a^)
Uncertainty as general term (basic uncertainty)	Outcome (O): yes/no Likelihood (L): yes/no Evaluation (E): yes/no	□⋄O ∨ □⋄L ∨ □⋄E It is necessary that it is possible to know *outcome*, *likelihood* or *evaluation* (or a combination of these)
Eventuation uncertainty (risk/chance)	Outcome (O): yes Likelihood (L): yes Evaluation (E): yes	□O ∧ □L ∧ □E It is necessary to know *outcome*, *likelihood* and *evaluation*
Evaluation uncertainty (ambiguity)	Outcome (O): yes Likelihood (L): yes Evaluation (E): no	□O ∧ □L ∧ □¬E It is necessary to know *outcome* and *likelihood*, but it is necessary to not know *evaluation*
Definiteness uncertainty (indefiniteness)	Outcome (O): yes Likelihood (L): (no) Evaluation (E): yes/no	□O ∧ □⋄L ˄ (⋄E >-< □¬E) It is necessary to know *outcome*, and it is necessary that it is possible to know *likelihood*, and it is possible to know *evaluation* or it is necessary to not know *evaluation*
Probability uncertainty (indeterminacy)	Outcome (O): yes Likelihood (L): no Evaluation (E): yes/no	□O ∧ □¬L ∧ (⋄E >-< □¬E) It is necessary to know *outcome*, and it is necessary to not know *likelihood*, and it is possible to know *evaluation* or it is necessary to not know *evaluation*
Contingence uncertainty (vagueness)	Outcome (O): (no) Likelihood (L): no Evaluation (E): yes/no	□⋄O ∧ □¬L ∧ (⋄E >-< □¬E) It is necessary that it is possible to know *outcome*, and it is necessary to not know *likelihood*, and it is possible to know *evaluation* or it is necessary to not know *evaluation*
Complete uncertainty (ignorance)	Outcome (O): no Likelihood (L): no Evaluation (E): no	□¬O ∧ □¬L ∧ □¬E It is necessary to not know *outcome*, *likelihood and*, *evaluation*

^a^Using a normal modal logic system or the K system, respectively; definition of propositional constants: O = knowledge of possible outcomes; L = knowledge of likelihood (of outcomes); E = knowledge of evaluation/characterization (of outcomes). Signs: □ = necessity, ⋄ = possibility, ¬ = Negation/not, ∧ = and, ∨ = or, >-<  = exclusive or (a >-< b = if it is the case that option a is true, option b cannot be true at the same time, but option b can be true if option a is not true; so both options can be true, but not at the same time.); yes = necessarily yes, no = necessarily no, yes/no = it is either possible yes or necessarily no, (no) = it is not necessarily no

The resulting category system is not intended to be an end in itself but to support decision-making. Therefore, it is important that the category system can be applied to *real-world* (research) examples. This allows the validation of the system itself (theory), and particularly *testing* its applicability and usefulness (practice). As such, the aforementioned example of gene therapy and somatic genome editing was selected as a ‘proof of concept’ for the category system.

If the category system is used in such a biomedical context, it is of paramount importance that the category system and its underlying concepts are (a) understandable by bioscientists, for example, researchers working in gene therapy and genome editing, and (b) that there are actual, identifiable examples of research that can accurately be assigned to the different concepts. In order to verify this, two bioscientists were introduced to the concepts and asked for suitable examples. Nevertheless, the main aim of this paper is to present a general category system. Therefore, the specific examples of uncertainty shown later will not be discussed in detail; their function consists only of showing the applicability of the category system to realistic situations in actual research.

## Definition and Dimensions of Uncertainty

Decisional uncertainty as a general term (or *basic uncertainty*) is defined as an epistemic state where there is no or only limited (*incomplete*) knowledge available. This incomplete knowledge about the consequence(s) can refer to a decision or an action (Han et al., 2011). More precise information relating to the following questions, either individually or together, is not known:

- What are the possible outcomes (*outcome dimension*)?^3^
- How likely are these outcomes (*likelihood dimension*)?^4^
- How should these outcomes be evaluated (*evaluation dimension*)?^5^

The knowledge items (a) to (c) are called “dimensions of states of uncertainty”.^6^ The epistemic state of uncertainty (as a general term) can relate to either the objective causes that generally limit human knowledge of these dimensions, or the subjective causes where the knowledge is insufficient (see “Causes of Uncertainties” section).^7^

**Fig. 1:**
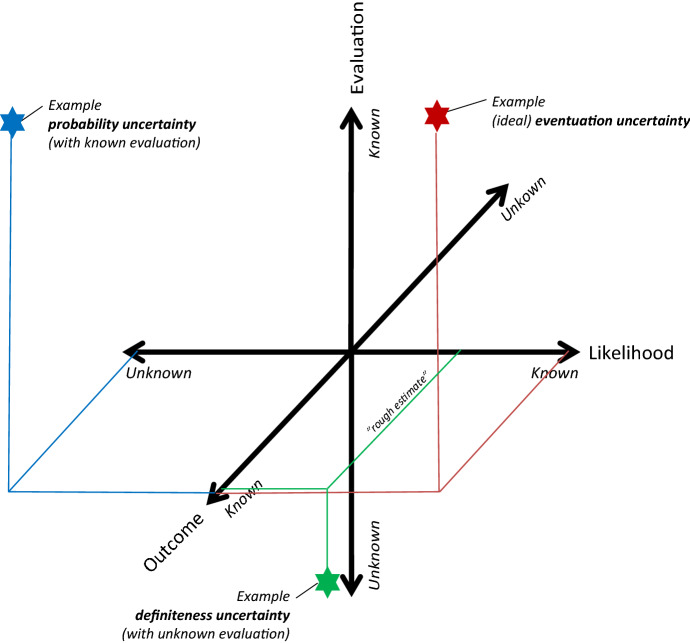
Three-dimensional Cartesian coordinate system of uncertainty (likelihood, outcome, evaluation)

*Specific* states of uncertainty can be understood as points in a three-dimensional Cartesian co-ordinate system (see Fig. 1; some inaccuracies are due to better displayability—however, this does not affect the heuristic function of the figure, which is to illustrate the interrelationships of the three dimensions).

## Specific States of (Decisional) Uncertainty

The extreme epistemic states at either ends of the continuum are *complete certainty* and *complete uncertainty*, respectively. Specific epistemic states of decisional uncertainty can be located between these two extremes and form types within the category system. Given the possibilities for combining the three uncertainty dimensions, the following list of seven individual specific epistemic states is incomplete; moreover, real cases will probably often blur the lines between the different epistemic states which are methodologically rather *ideal types*. The presentation of certain combinations in this paper is specifically done in order to point out those states that are deemed especially important, both here and based on the literature reviewed.

These seven specific states of decisional uncertainty, where the complete uncertainty mentioned previously is deliberately included as well, are explained in detail below (an overview, including expressions in modal logic, is shown in Table 1). Examples from research with gene therapy or genome editing that could correspond to the different states are listed in Table 2.

**Table 2:** Examples in gene therapy/genome editing

States of uncertainty	Examples in gene therapy/genome editing
**Eventuation uncertainty** (risk/chance) > 2 known outcomes (X, Y) Evaluation is known Likelihood is known	An example of this uncertainty, albeit not without problems (see below), is the adverse outcome of clonal dominance (which can cause leukaemia) and insertional mutagenesis [EVALUATION] after lentiviral haematopoietic stem and progenitor cell (HSPC) gene therapy for X-SCID (X-linked Severe Combined Immunodeficiency) or WAS (Wiskott-Aldrich syndrome) patients (Aiuti et al., 2013) [OUTCOME]. The probability of this risk is estimated based on existing clinical studies (there are no cases where insertional mutagenesis has happened so far) [LIKELIHOOD], which, however, only have small sample sizes (5–10 patients/study), thus, reducing statistical validity^a^ *Possible causes of uncertainty: State of evidence (quantity and quality of existing research)*
**Evaluation uncertainty** (ambiguity) Outcome is known Likelihood is known Evaluation is unknown	Evaluation uncertainty is seldom encountered in early clinical research, because preclinical studies already indicate whether biomedical products and their intended therapeutic effects have to be deemed *good* or *bad* for future patients. However, in the example of genetically modified mesenchymal stem cells (MSCs) [OUTCOME AND LIKELIHOOD], it is not unequivocally clear to what extent the genetic manipulation improves or even worsens the therapeutic MSC functions [EVALUATION] in patients suffering from various diseases such as cancer, especially, in the long term (Mosallaei et al., 2020) *Possible causes of uncertainty: Structure (value pluralism), State of evidence (quantity and suitable type of existing research)*
**Definiteness uncertainty** (indefiniteness) Outcome is known Likelihood knowledge is limited, tentative Evaluation can be known, but does not have to be known	An example of definiteness uncertainty is the knowledge concerning the rate and time frame of immune reconstitution [OUTCOME] after gene therapy for X-SCID, which renders infants extremely vulnerable to infections with various pathogens [EVALUATION]) (Hacein-Bey-Abina et al., 2014). This is uncertain due to the variability in kinetics and efficiency of the therapy between treated patients [LIKELIHOOD] *Possible causes of uncertainty: Structure (complexity, inherent randomness/unpredictability)*
**Probability uncertainty** (indeterminacy) Outcome is known Likelihood is unknown Evaluation can be known, but does not have to be known	This uncertainty can be observed in genotoxic [EVALUATION] insertional mutagenesis as an outcome of CRISPR/CAS9- and ZFN-mediated off-target cleavage in HSPC, for example, CCR5, disruption (Xu et al., 2019) [OUTCOME]. Although the mechanisms that lead to genotoxicity are known, and off-target cleavage in, for example, tumour suppressor genes and insertional mutagenesis are both known to be rare, it is not possible to know currently whether this is relevant in a clinical setting, i.e. whether there will be genotoxicity [LIKELIHOOD]. However, as such genotoxic off-target effects have been observed in retroviral vector-treated patients, a comparable phenotype of genotoxicity insertional mutagenesis could be possible by disrupting tumour suppressors *Possible causes of uncertainty: State of Evidence (quantity and suitable type of existing research)*
**Contingence uncertainty** (vagueness) Outcome is tentative Likelihoods are unknown Evaluation can be known, but does not have to be known	An example of contingence uncertainty is the outcome of CCR5 mutations in CRISPR/CAS9-manipulated babies with the goal of immunizing them from HIV and consequent negative effects resulting from this particular *wanted* mutation (Wang & Yang, 2019) [OUTCOME]. It is known that the common homozygous CCR5Δ32 mutation in a small population of Europeans leads to no major adverse events (besides susceptibility to the West Nile Virus). However, it cannot be ruled out that the broader spectrum of CRISPR/CAS9-induced CCR5 mutations could lead to negative effects [OUTCOME AND EVALUATION], and no probabilities can be estimated [LIKELIHOOD] *Possible causes of uncertainty: Structure (complexity), State of evidence (quantity of research)*
**Complete uncertainty** (ignorance) Outcome is unknown Likelihood is unknown Evaluation is unknown	Complete uncertainty can be seen regarding the outcome and its effects (and whether they are positive or negative) [EVALUATION] of off-target mutations in CRISPR/CAS9 CCR5-manipulated babies (Wang & Yang, 2019), i.e. of *not intended* mutations at DNA sequences that are very similar to the target DNA sequence of the intervention, but not identical [OUTCOME]. There is currently absolutely no knowledge of whether CRISPR/CAS9-manipulation of whole human body cells may lead to severe effects in babies in the long term [OUTCOME, LIKELIHOOD, EVALUATION] *Possible causes of uncertainty: Structure (complexity, novelty), State of evidence (quantity and quality of research), Application (knowledge from patients)*

^a^Given the evidence mentioned, it can be objected that in this case, the probability cannot (really) be known. However, the example still comes closest to eventuation uncertainty compared to other examples, and it is at least not the case that there is nothing at all on which one could make a probability assumption. So, *prima facie*, it can be categorized into eventuation uncertainty, though we concede that depending on how evidence is appraised and considered ‘sufficient’ for a given context, it could also *post factum* be categorized to e.g. definiteness uncertainty

### Eventuation Uncertainty (Risk/Chance)

Eventuation uncertainty is defined as an epistemic state where one knows which outcomes are possible, what the likelihood of these outcomes is (“know the odds”, Wynne, 1992, p. 114) and how one should evaluate these outcomes.^8^ The uncertainty here relates to the difference between the known possible consequences (outcomes) and the consequences that actually occur (i.e. eventuation). It can be assumed that at least this kind of uncertainty is present in any situation where a decision is made. One can remember the example of the fair die to illustrate this. If one is rolling a die, it is clear what the outcomes can be and what the associated likelihoods are. But even here, it is not possible to forecast what outcome will occur before the die is rolled. This state of uncertainty cannot be overcome with additional knowledge since it is always present—with the possible exception of a (theoretically) fully deterministic system where there is certainty that a specific consequence will follow (Wynne, 1992). (However, regarding especially early clinical trials, such a situation where there is a certainty about specific consequences will probably never occur, therefore, this situation will not be discussed further). In addition, eventuation uncertainty differentiates between knowing the theoretical outcomes and the outcomes as they happen in (a future) reality; it emphasizes the uncertainty relating to which of the possible outcomes is going to happen. Based on the current state of research, for example, an intervention of a vaccination against measles could lead to (generally successful) immunization without side-effects with a 95% likelihood or could lead to (generally successful) immunization with a measles-like skin rash and fever with a 5% likelihood (Robert Koch Institut, 2016).^9^ It is clear which outcome is more likely, but even with such a high likelihood, there is no certainty as to what is actually going to happen (Djulbegović, 2007; Wöhlke et al., 2019).

### Evaluation Uncertainty (Ambiguity)

Evaluation uncertainty is defined as an epistemic state where one knows which outcomes are possible and what the likelihoods of these outcomes are. However, the evaluation of the possible outcomes is uncertain because there is insufficient knowledge concerning how to interpret the outcomes and judge them (i.e. how important they are, what they mean, for example, for patients and their health, and whether, on the whole, they are *good* or *bad* outcomes) (Stirling & Scoones, 2009). This epistemic state can also be labelled ambiguity.^10^

A simple example to illustrate this can be found when playing board games. If one is learning a new game and begins to understand the rules, one knows which outcome is caused by which action, i.e. a specific move on the game board; however, it can be very difficult to interpret the events and to decide whether a particular move would be a *smart* move. (In this example, this lack of knowledge can be overcome by playing the game regularly, and also asking more experienced players.)

Evaluation uncertainty in biomedical contexts, therefore, refers to questions such as whether the physiological effects of, for example, a drug would be medically or ethically *good* or desirable by considering the possible benefits and unwanted side effects. Evaluation generally has to refer to *evaluation standards* (which define what is *good* in relation to a specific goal, task or function; Fogelin & Sinnott-Armstrong, 2005), for example, standards related to biomedical science, clinical research or health economics, for such considerations. Thus, evaluation uncertainty may (also) be related to not knowing which standards are relevant or applicable, or whether the evaluation standard itself is sufficiently robust and thought through (see, for instance, the debate about QALYs, *quality-adjusted life years*, as a health economics measure; e.g. Bognar, 2015). It can also be the case that regarding one standard, for example, on the level of biological mechanisms (biomedical science), evaluation of the respective outcomes is sufficiently known, but not regarding other standards, such as the clinical outcome level or especially the psychological or social level. It is still debatable (not decisively known), for example, how cochlear implants for deaf children should be evaluated, because even when the child learns to interpret the signals from the implant (which can also fail), the deaf community considers the whole intervention as a disruption of their own culture.

Ambiguity may also occur in other specific states of uncertainty. These are described below, including those where the outcome is not yet known.

### Definiteness Uncertainty (Indefiniteness)

Definiteness uncertainty is defined as an epistemic state where one knows which outcomes are possible, but the likelihood of the occurrence of each outcome is not *definitely* known. This is, at first, in line with the common understanding of uncertainty in the aftermath of Knight's approach. However, for this particular epistemic state, it is more correct to say that it may be possible to provide an orienting estimate about how high or low the likelihood is (e.g. “not higher than 60%”), but not the exact percentage.^11^ Regarding the context, this uncertainty can have a different focus. The focus in daily life may lie more often on the outcome itself, and more on the likelihoods in a research context. The two following examples can illustrate this. Imagine a scene in a café. If one asks the waiter to bring coffee *or* tea, one is (*ceteris paribus*) determining the possible outcomes of a situation, and one still cannot know which hot drink one will finally get. Furthermore, it cannot be said which likelihoods the specific outcomes (tea, coffee) have. However, one may guess which hot drink will be more likely to be served based on, for example, previous knowledge. Perhaps the place is known for the best coffee in town, or it is known that this waiter has a special favourite that he/she likes to offer.

An example in which the focus shifts from the outcome to the likelihoods can be found in drug development. There are observed outcomes and first estimations about the appropriate likelihoods in the transition phase from animal testing to human testing. However, it is possible that an effect that was observed in the animal testing has a different intensity in humans. This means that likelihoods derived from the observations in animal testing cannot be transferred without limitations (i.e. not exactly) to the application in humans. However, one is able to estimate the likelihood based on data existing already.

### Probability Uncertainty (Indeterminacy)

Probability uncertainty can be understood as an escalation of the definiteness uncertainty mentioned above. As is the case with definiteness uncertainty, the possible outcomes are known. The difference between definiteness and probability uncertainty is that in the latter there is necessarily no available knowledge of the likelihood, not even in the form of a rough estimate.^12^ This state of uncertainty can often be found in new or *idiosyncratic* situations where there is no basis (or currently no basis) for even rough estimates of likelihoods (e.g. no comparable cases in the past, or the events are causally complex due to having many, probably also in part unknown causes). Regarding a revolution/insurrection in a *failed state*, for example, there can be the failure or success of the insurrection (in relation to the goals of revolutionaries) as known possible outcomes. But it is not possible to determine likelihoods, not even rough estimates, because of the possibility of so many unpredictable events that occur in the process of an insurrection, or due to the fact that there are never exactly equal situations (based on which one could, at least, estimate likelihood).^13^ One may argue that a particular event, for example, the failure of the insurrection, is *more likely* than the other (given certain political or military conditions), but one will not be able to determine *how* likely (compared to the other outcome(s)).

The importance of differentiating between *definiteness uncertainty* and *probability uncertainty*, especially in research contexts, can be illustrated by the difference in what it means for a decision maker to be in one of these two states of uncertainty. In the *definiteness uncertainty* example, the likelihood cannot be exactly determined, but the estimates available still help to decide whether, for example, a drug should be tested in humans or not. Knowing the likelihood is important knowledge for minimizing the potential harm to the participants of a study. If the observation in animal testing shows that, for example, only 2% of the animals die or suffer side effects from the drug, the uncertainty for participants of the study is likely to be lower than without such data. In the state of *probability uncertainty*, this knowledge is not available, therefore, the uncertainty for the study participants is much higher.

### Contingence Uncertainty (Vagueness)

It is not necessary to have any knowledge of outcomes for contingence uncertainty, although it is *possible* to have knowledge—albeit rather tentative, i.e. there is no certainty about *what* the possible consequences (outcome) could be. In summary, there is a “range of potential futures” (Djulbegović, 2007, p. 83), but it is not known which futures are realistic possibilities. Thus, assertions about the outcomes of an action remain *vague*.^14^ A situation where this uncertainty can be found appears for everyone after graduation. One needs to decide what one would like *to do* for the rest of one’s life. Even if one has an idea about what one wants to study, for example, one cannot predict what the future will look like. The exact consequences of one’s decision cannot be foreseen, since the consequences depend on many factors (e.g. where one will live and whom one will meet, whether one will stay healthy, etc.), some of which may also presumably be unknown. An example in the context of biomedicine could occur when transitioning from basic research to animal testing of *promising* substances. There, it will normally be uncertain, for example, whether the substance tested will (finally) lead to a useful and safe drug, and how effective it will be exactly (various positively evaluated outcomes are conceivable). Additionally, it is uncertain whether—despite all animal testing—it may lead unexpectedly to severe adverse effects when applied to humans (various negatively evaluated outcomes are conceivable, e.g. *kidney failure*). It could also be possible that the substance tested will show no effectiveness in later preclinical or clinical phases, or will only have a clinically negligible effect.

### Complete Uncertainty (Ignorance)

Complete uncertainty is characterized by not knowing anything regarding the three dimensions of uncertainty. Thus, there is no knowledge of the outcome, the likelihood and the evaluation in this epistemic state. In contrast to contingence uncertainty, the lack of knowledge in the state of complete uncertainty is more fundamental and is not limited in any way. Not only is the outcome unknown, it is also not clear how an outcome would be identified, what could influence it or under what circumstances it would be influenced.

However, it is important to emphasize that complete uncertainty does not have to extend to the entirety of the object of research. It can be any detail that influences the outcome without knowing that it has such an effect (Wynne, 1992).^15^ The understanding of ignorance or complete uncertainty referred to in this paper does not presume a wilful decision of ignorance, but describes an epistemic state where unawareness (or even the inability to be aware) is a result of objective or subjective factors relating to the relevant knowledge. In this regard, ignorance can be an “invisible uncertainty” because its existence is unknown. Thus, it can be debated whether complete uncertainty about all aspects is a *realistic* epistemic state, particularly in modern clinical research (cf. Djulbegović, 2007), since it is not often that there are not even slight assumptions based on at least some experience, comparisons to comparable cases, physiological or biochemical theories or just expert opinions. Nevertheless, even if complete uncertainty is often not a realistic epistemic state, it is a useful concept as one end of the continuum between *knowing* and *knowing nothing*.

## Causes of Uncertainties

The authors define three main categories of causes of uncertainties. This categorization is oriented to different facets of research, its process and its reception, inspired by Han et al., (2011). The first category looks at the *structure of the object of research*, the second at the *state of the evidence* of the research and the third looks at the *individual handling of the research and already existing knowledge*.

### Structure

The structure of the object of the research has an important influence on the research itself and determines the possibility of acquiring scientific knowledge. In nursing, for example, the structure and process of care are aspects that Han et al., (2011, p. 835) call “system-centred sources”. These form the system and framework in which nursing takes place. Understanding system-centred sources or the structure of the object of the research is more general and not limited to biomedical research. A variation of the categorization of Han et al. (2011) which includes at least two main causes is presented here. One of the main causes of uncertainties found in the structure of research belongs to the object itself. Difficulties in reaching certainty arise due to complex systems which are not easy to explore or show inherent randomness (not reducible even in principle), natural variability and unpredictability, making it impossible to fully explore and understand them (Renn et al., 2011; Wynne, 1992). Another cause could be the relationship between the model of the research and reality. Difficulty in depicting the more complex world in a subcomplex model could cause uncertainties, where there are uncertainties about whether it functions the same way in reality as in the model (Wynne, 1992). Similarly, a cause of uncertainties that refers particularly to the dimension of evaluation is value pluralism (Renn et al., 2011). This pluralism causes uncertainties because there is more than one possible evaluation standard that could be applied to the outcomes, and is, thus, a relevant cause of ambiguity. The second main cause of uncertainties is the novelty of a phenomenon: if a phenomenon is newly discovered, it is impossible to compare the results with the results of other research or make prognoses based on existing knowledge.

### State of Evidence

The state of the evidence refers to research that has already been undertaken. Even where there is a lot of research available, uncertainties cannot automatically be ruled out. This is because of the quality of the research, for example, whether the design of a study fits the object pursued or the arguments given are valid and sound. Furthermore, the accessibility of the results is decisive in the question of uncertainties. It is clear that the *individual researcher* depends on access to existing and, at best, good research to minimize uncertainties in their own research. Another aspect that causes uncertainties is found in the interpretation (Wöhlke et al., 2019) and evaluation of data. Data can be interpreted in different ways, and the evaluation of data in particular can differ depending on the person doing the evaluation.

### Application

Uncertainties can further arise because of the researcher’s individual incomplete knowledge. This incompleteness is not caused by the fact that there is no knowledge available, but by the fact that *the individual researcher does not know that this knowledge is available*. This is not only restricted to the research available, but also includes important information that could be obtained from patients/research subjects or their relatives, such as information about the subject’s condition, their personal values or medical history. In abstract terms, knowledge from research or other sources (e.g. clinical context) may be incomplete and lead to uncertainties.

The three main causes of uncertainties proposed show some significant differences. One of these is the fact that the causes of uncertainties in the first category can possibly not be overcome. In the case of the inherent randomness of an object of research, for example, it appears to be objectively impossible to reach pragmatic certainty. This circumstance differs in part regarding the other two categories. The quality, quantity or accessibility of research could, in principle, be good enough that it no longer causes (considerable) uncertainties. In addition, individual knowledge could be sufficiently good that it also does not cause uncertainties. These causes, however, produce uncertainties due to mainly individual, but also structural problems.

## Outlook

Considered from a *theoretical perspective*, the category system and its underlying concept of three different dimensions of uncertainty is sufficiently general to capture uncertainty epistemologically in different settings. The particular epistemic states of uncertainty are systematically derived (and explained) through modallogical terms that depict the three dimensions, thus are theoretically replicable. This derivation also allows for a more precise identification of the relevant incomplete knowledge which constitutes particular uncertainty, as well as for an explicable increasing escalation of the gravity of uncertainty (by adding more and more incomplete knowledge from the three dimensions). The fact that the epistemic states often approximate different types of uncertainty described in the literature is not a weakness, but rather a strength: On the one hand, it shows the *connectivity* of the category system to existing discourses; on the other hand, the concept of the three dimensions has the potential to transparently *reconstruct* the types of uncertainty already described by means of the combination of the three dimensions. Both gives an additional coherentist justification for the descriptions of epistemic states, which is important if the category system is to be applied practically.

This is because, from a *practical perspective*, the aim is to apply the category system to early clinical trials in particular. For this purpose, the epistemic states of the category system allow to express the actual uncertainty. The category system has also been tested for its application in such settings, using the fitting example of gene therapy and genome editing, with the potential to complement an already developed ‘risk matrix’ for RBA in this area (Bitterlinger et al., 2022). That the category system can be applied to real applications and understood by life scientists is a fundamental precondition for a more adequate ethical analysis within RBA based on a better understanding of the epistemic state applicable. The category system may also invite one to think about benefits more as *chances*, occurrences that, in principle, could also be expressed in probabilities (where epistemically possible), alleviating the problem that *risk* in RBA is (often) a combination of likelihood and harm, while *benefit* is rather only an *anticipated fact* or a desirable state of the world.

Furthermore, differentiating between states of (decisional) uncertainty and causes of uncertainties upholds the separation of two sometimes conflated categories, and improves the detailed analysis of the state of uncertainty with which one is confronted. This is crucial for assessing the *weight* of the uncertainty (i.e. can it be overcome or not, and if yes, how?), which should also be honestly communicated to study participants/patients.

However, the exact ethical implications of the different states of uncertainty, and possibly also their causes, need be explored in more detail in future. Accordingly, it will be necessary to take a closer theoretical look at different decision-logic models (e.g. effectuation logic vs. causation logic), rules or principles for decision-making under uncertainty (e.g. bootstrapping, precautionary principle) and ethical principles (e.g. individual benefit and social/scientific value vs. harm). Therefore, *test questions* for each state of uncertainty could be introduced in a possible further paper to help identify in which state one is. As soon as there is consensus between the researchers (or other relevant decision makers) which epistemic state prevails, various options that support decision-making could then be offered, such as which decision logic and existing RBA process would be appropriate. Additionally, different ethical criteria could be used to consider whether or not an (early) clinical trial should be conducted or not, or to what to pay special attention. In addition, advice could be given on what information should be included in the informed consent document processes, or how best to communicate the uncertainty about harms and also benefits that exists. Clues for how to mitigate the state of uncertainty by helping to identify the causes of the uncertainty could be additionally included.

However, this can only work in co-operation with bioscientists who regularly, among other things, plan and conduct early phase trials, as they can deliver concrete instances of research that can be analysed with the category system developed here, suggest how they would react to the given uncertainty and discuss this within the context of research ethics. On this basis, RBA and informed consent documents, especially, but not limited to gene therapy/genome editing, can be improved using the concepts developed here and in the future.

## Footnotes

1 For example, ‘uncertainty of demarcation’ (Hansson, 1996), where decision makers disagree on *what* the decision should be about, is less relevant in early clinical research than in politically contested decision-making. This is because the decision is rather unambiguously about (not) initiating/conducting the study.

2 The authors acknowledge that there are different positions on this issue, especially when subjective, Bayesian probabilities are considered, not just objective probabilities in, for example, a frequentist interpretation. Still, because it is debatable, a probable *presupposition* that ‘classic’ RBA can be applied to *every kind of uncertainty* is critically regarded (see also Bradley & Drechsler, 2014).

3 Here, “outcome” is used in a rather wide meaning as ‘consequences of an intervention’ (including changes only observable in, for example, pharmacokinetics or gene expression), not only meaning *clinical outcomes* based on defined endpoints. This is because the latter often already incorporates evaluative meaning (e.g. ‘quality of life’ as an endpoint) that the authors want to deliberately separate in their account of uncertainty dimensions. That being said, “outcome”, as in decision theory, can be understood as a result of the occurrence (existing or not) of an act (that an agent can control) and an event as a set of states of the world (that an agent cannot control). The understanding of “possible outcomes” (as, for example, ‘all logically possible outcomes’) is dependent on the concrete method/approach used.

4 “Likelihood”, similar to “outcome”, is used in a rather broad understanding. It depends on the concrete method/approach chosen whether it is interpreted as expressing objective probabilities (frequentist or propensity accounts) or subjective/Bayesian probabilities. It is only important that there can be a measurable likelihood (how probable is it, or is it expected, that a certain outcome occurs?), however defined.

5 Or more exactly: Should these outcomes (or, at least, the hypothetically assumed outcomes) be characterized/evaluated as harms or benefits, and how severe are the harms and how beneficial the benefits (e.g. outcomes regarding their effect on a patient’s overall well-being or merely regarding certain effects on a patient’s body in the long term). Additionally, one can differentiate between mere *clinical/medical* outcomes and *social* outcomes. The evaluation of social outcomes is often more characterized by uncertainty than the evaluation of clinical outcomes; still, evaluation uncertainty is at least *conceivable* for both kinds of outcomes.

6 The authors do not claim that the dimensions are independent of each other; of course, the evaluation of an outcome is (also) dependent on the *nature* of that very outcome. However, it is possible to differentiate our knowledge (or lack thereof) of the various dimensions logically and epistemically, and this can have practical importance, for example, where there is no agreement on evaluation standards.

7 Out of completeness, a fourth dimension in any state of uncertainty could be mentioned; this could be named the *eventuation dimension*. Eventuation addresses the question of whether the expected outcome will actually be reality after the action, irrespective of whether it has a high or low probability; exemplarily, when having a 50% likelihood that tails will be on the upper side when tossing a coin, it is, nonetheless, not known whether tails or heads will *actually* be on the upper side until the very moment the coin has landed on the ground. This dimension will be neglected in the remainder of this paper due to its constant presence but little practical effect for *real-world* decision-making.

8 Eventuation uncertainty is associated with the established term ‘risk’. As Möller et al., (2006, p. 421) indicate, there are several established concepts of ‘risk’, e.g. “an *unwanted event* which may or may not occur”, the *cause* of such an unwanted event, or its *probability*. Most authors (Genske & Engel-Glatter, 2016; Hunt, 1994; Wynne, 1992) define ‘risk’, following Knight (1921), as a state where one knows the possible outcomes as well as the likelihoods of these outcomes. Walker et al. (2013) call this “level 2 uncertainty” and characterize it as those uncertainties that can (still) be described statistically. Hansson (1996, p. 376) considers it as the first degree of ‘uncertainty of consequences’, as “decision making under known probabilities”. (Note that ‘risk’ is associated with outcomes that are evaluated as being harmful or undesirable, while ‘chance’ refers to outcomes that are beneficial or desirable.) The difference between the established definition and the definition of eventuation uncertainty is that, in the latter, it is also necessary to know how the outcomes would be evaluated.

9 One could argue that these numbers can be doubted on the background of what may be called *evidentiary* uncertainty or, up to a certain degree, ‘uncertainty of reliance’ (doubt whether the information from others is reliable; Hansson, 1996), as distinguished from decisional uncertainty. There is a difference between *knowledge* and the *trust* we may have in this knowledge, or how high the ‘severity of uncertainty’ is (“[…] the *difficulty* the agent has in making a judgement about the prospects they face, a feature that depends on the amount of judgement-relevant information that is available to them” or “how coherent this information is […]”, (Bradley & Drechsler, 2014, p. 1231); the evidence available, especially in biomedical contexts, is, of course, no *ultimate truth*. Such uncertainty would be a kind of a meta-uncertainty (or is “orthogonal” to other types of uncertainty, e.g. about possible consequences, as Bradley & Drechsler, 2014, note for their own typology). It cannot be practically ignored in the end but has to be differentiated from epistemic states on the decisional level.

10 The established term ‘ambiguity’ is used differently in the literature. Ellsberg (1961) and Politi et al. (2007), for example, use it to describe a state where evidence is available, but the quality (strength or validity) of the evidence is untrustworthy. In the terminology used here, this is rather an aspect of ‘evidentiary uncertainty’ (see footnote 10). Stirling (2009) and Genske and Engel-Glatter (2016), following Stirling and Scoones (2009) and Funtowicz and Ravetz (1990), on the other hand, use it in a similar way to that proposed here, i.e. *evaluation uncertainty*. Walker et al. (2013) also refer to the problem of how to value the desirability of various outcomes, although they see this more as an (additional) aspect of their “level 4” and “level 5” uncertainties. Bradley and Drechsler (2014) subsume the problem under the heading of ‘normative uncertainty’ or ‘ethical uncertainty’, Hansson (1996) described it as ‘uncertainty of values’ (which, however, is focused more on the lack of knowledge about the—current or constant—values of the decision makers). In sum, these definitions focus more on the difficulty of evaluating the outcome than on the valuation of evidence.

11 Aspects of this epistemic state can be recognized in what Walker et al. (2013) call “level 3 uncertainty”, where there can be several scenarios about the future world that can be ranked on the basis of *perceived likelihoods*, but an assignment of (unique) probabilities is not possible. It is also comparable to Hansson’s (1996, p. 376) “decision making under incompletely known probabilities” as part of ‘uncertainty of consequences’.

12 This state of uncertainty, where one knows the outcome but not the likelihood of an action, is well-known in the literature, although it is termed differently by various authors (e.g. Genske & Engel-Glatter, 2016; Hunt, 1994; Nida-Rümelin et al., 2012). It is comparable (although not entirely congruent) to the “level 4 uncertainty” of Walker et al. (2013), where there are several plausible alternatives, but it is not possible to rank them even with perceived likelihood. It is one aspect (but does not correspond to it exactly) of ‘empirical uncertainty’ (“uncertainty about what *is* the case”) by Bradley and Drechsler (2014, p. 1229), and of “decision-making under unknown nonzero probabilities” as part of ‘uncertainty of consequences’ (Hansson, 1996, p. 376). Using the term *indeterminacy* seems appropriate, since it accentuates only a lack of quantitative information (likelihood) and not qualitative information (i.e. which outcomes are possible).

13 Although it has to be acknowledged that here using subjective probabilities *could* be a solution. Nevertheless, it might be questionable how *good* such an estimate is in view of the difficulties of estimating probabilities which have been mentioned.

14 Where contingence uncertainty as ‘vagueness’ has been described as an epistemic state in the existing literature, it has been named differently, for example, as ignorance or ambiguity (e.g. Genske & Engel-Glatter, 2016; Stirling, 2009), which, in the terminology used in this paper, refer to other epistemic states. However, there are slight similarities with Walker et al.’s (2013) “level 5 uncertainty”, in the sense that at least what is not known is known (recognized ignorance), i.e. one knows that one does not know the outcomes, nor their probability exactly. ‘Modal uncertainty’ (“uncertainty about what is possible or about what could be the case”) by Bradley and Drechsler (2014, p. 1229) also goes in a similar direction, but they delineate it typologically quite differently (it is part of the ‘nature’ of a judgment, i.e. whether it is modal, empirical or normative). The closest to describing this condition is Hansson’s (1996, p. 376) “decision making under unknown possibilities” as part of ‘uncertainty of consequences’.

15 Brian Wynne called these “indeterminate actors” and emphasized that anything from a stone to a manager could be an “indeterminate actor” (1992, p. 117).
